# Unexplained Intra-abdominal Abscess: A Case Report and Literature Review

**DOI:** 10.7759/cureus.89011

**Published:** 2025-07-29

**Authors:** Yatin Srinivash Ramesh Babu, Alison Thornton, Ramon Gil, Jeffrey Snow

**Affiliations:** 1 Medicine, Dr. Kiran C. Patel College of Osteopathic Medicine, Nova Southeastern University, Fort Lauderdale, USA; 2 General Surgery, Hospital Corporation of America (HCA) Florida Northwest Hospital, Margate, USA; 3 Colorectal Surgery, Memorial Healthcare System, Hollywood, USA

**Keywords:** geriatrics population, intra-abdominal, intra-abdominal abscess, surgical case reports, trauma

## Abstract

This is the case of a 65-year-old female patient with chronic obstructive pulmonary disease (COPD) who presented following a fall and was found to have a humeral fracture. Initial evaluation showed sepsis of unknown origin, leukocytosis, and elevated inflammatory markers. Persistent abdominal pain prompted imaging studies, which showed multiloculated intra-abdominal abscesses. Ultrasound-guided drainage yielded *Streptococcus anginosus and Bacteroides fragilis*. Antibiotics, along with percutaneous drainage, resulted in a good clinical response. Follow-up imaging revealed a decrease in the abscess size, with no evidence of a fistula. This case highlights the diagnostic challenges of intra-abdominal abscesses and the importance of timely imaging in patients presenting with sepsis and nonspecific symptoms.

## Introduction

Intra-abdominal abscesses (IAAs) are localized accumulations of pus found in the peritoneal cavity, typically arising from infections that compromise the body's natural defenses. These abscesses generally occur from localized inflammation, tissue death, or microbial infection [1,2]. Although appendicitis, diverticulitis, inflammatory bowel disease (IBD), and perforated organs are among the primary causes, IAAs can also arise after surgery or because of trauma [3,4].

Clinical features vary widely, often including fever, abdominal pain, nausea, vomiting, and leukocytosis. On the other hand, non-specific symptoms may complicate diagnosis in atypical cases [5]. The IAAs are often associated with other factors, such as diabetes, malignancies, and immunosuppression, which increase either susceptibility to infection or impair healing processes [6].

Due to their varied origins, the differential diagnosis for IAAs is extensive, including gastrointestinal perforations, neoplasms, hematomas, and retroperitoneal abscesses [7]. This complexity underscores the importance of imaging, especially contrast-enhanced computed tomography (CT), as a reliable tool for verifying the diagnosis and directing treatment [8,9].

This case report discusses a female patient in her mid-60s with a history of chronic obstructive pulmonary disease (COPD) and failure to thrive following a mechanical fall at home. Her case was complicated by an atypical presentation of a multiloculated IAA with an unknown etiology, underscoring the diagnostic challenges and emphasizing the crucial need for a thorough evaluation.

## Case presentation

**Figure 1:**
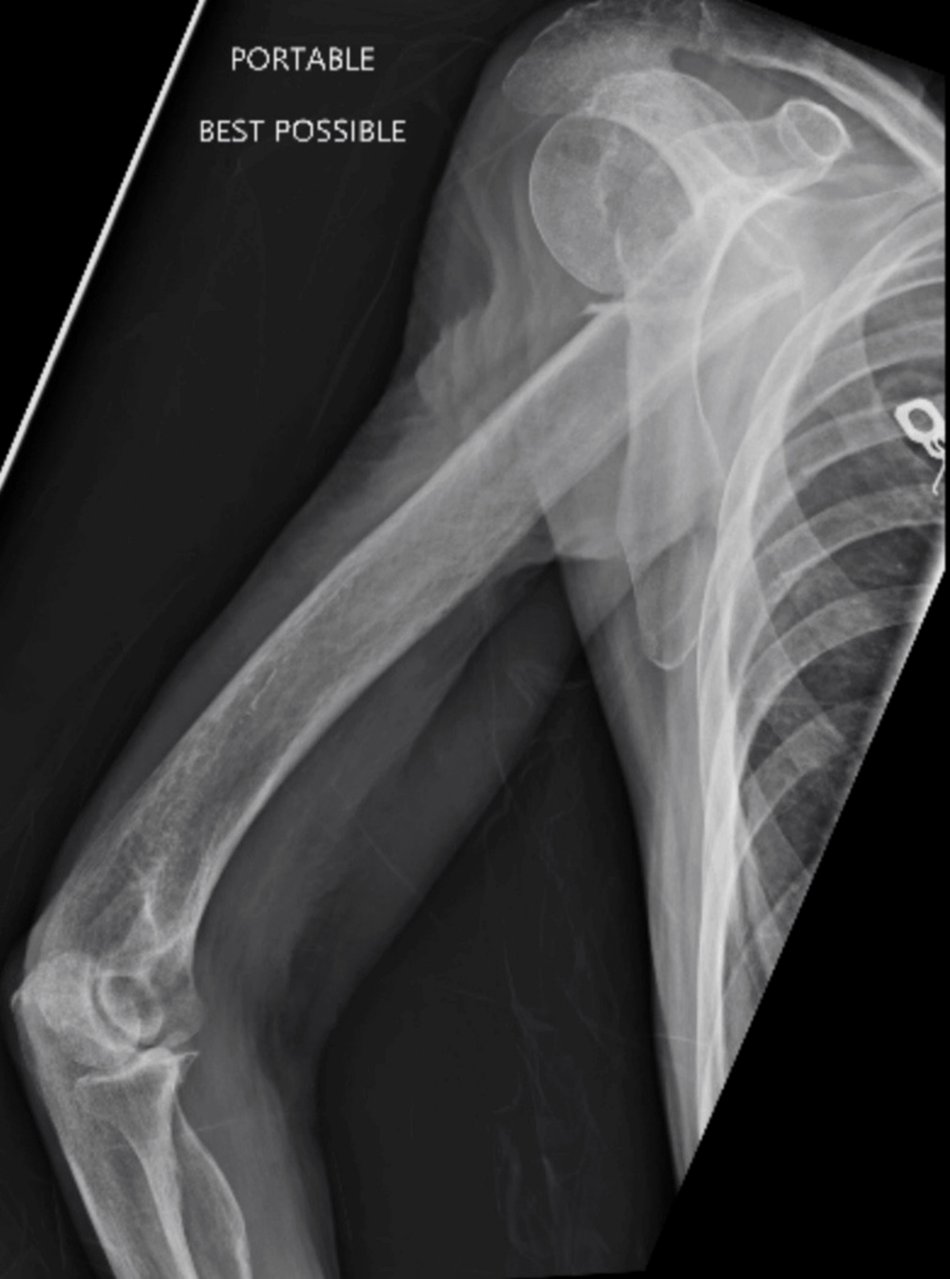
Humeral X-Ray. Right proximal humeral fracture at the surgical neck with posterior displacement of the distal segment.

**Figure 2:**
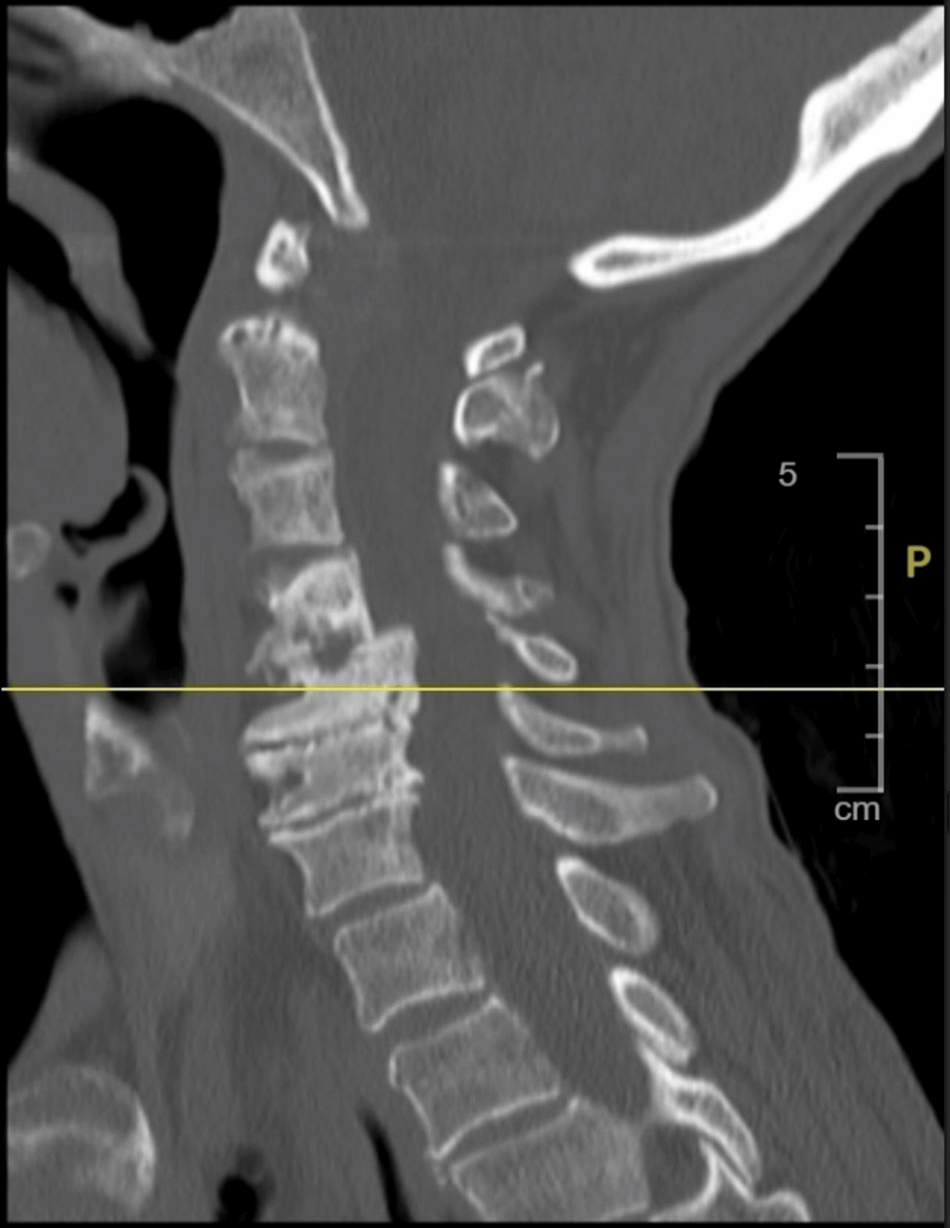
CT cervical spine. No acute fracture of the cervical spine noted. Multilevel advanced degenerative changes are most significant at C4-C5 level.

A female patient in her mid-60s with a history of COPD presented to the Emergency Department after a mechanical fall at home. She reported pain in her right arm and had limited recall of the fall. She denied associated symptoms such as cough, shortness of breath, nausea, vomiting, or diarrhea. Physical examination revealed significant tenderness in the right upper extremity. Initial imaging identified a right humeral fracture with posterior displacement and a left humeral fracture (Figure 1). Cervical spine imaging revealed severe stenosis (Figure 2). Laboratory analysis was notable for hypokalemia (2.6 mmol/L), corrected in the Emergency Department. Orthopedic consultation was obtained for fracture management.

On arrival at the Emergency Department, the patient’s vital signs were 98.2°F, blood pressure 119/85 mmHg, heart rate 101 bpm, and respiration rate 18 breaths per minute. Laboratory work, including serum chemistry, complete blood count, and urinalysis, is shown in Tables 1, 2.

**Table 1:** Patient’s Complete Metabolic Panel on Admission (H)=High value, (L)=low value

Test	Result	Reference Range
Sodium	140 mmol/L	135-145 mmol/L
Potassium	2.6 mmol/L (L)	3.5-5.2 mmol/L
Chloride	99 mmol/L	95-110 mmol/L
Carbon dioxide	23 mmol/L	19-34 mmol/L
Blood urea nitrogen	33 mg/dL (H)	6-22 mg/dL
Creatinine	0.47 mg/dL	0.43-1.13 mg/dL
Estimated glomerular filtration rate	>90	≥90
Glucose	128 mg/dL	70-110 mg/dL
Lactic acid	1.0 mmol/L	0.4-2.0 mmol/L
Calcium	10.7 mg/dL (H)	8.4-10.2 mg/dL
Magnesium	1.8 mg/dL	1.6-2.4 mg/dL
Total bilirubin	1.7 mg/dL (H)	0.1-1.2 mg/dL
Aspartate aminotransferase	48 Units/L (H)	10-40 Units/L
Alanine aminotransferase	26 Units/L	10-60 Units/L
Alkaline phosphatase	302 Units/L (H)	20-130 Units/L
Troponin I	<0.012 ng/mL	0.000-0.034 ng/mL
N-terminal pro-B-type natriuretic peptide	1280 pg/mL (H)	0-217 pg/mL
Total protein	8.0 g/dL	5.5-8.7 g/dL
Albumin	3.9 g/dL	2.8-5.5 g/dL

**Table 2:** Patient’s Complete Blood Count on Admission (H)=High value, (L)=low value

Test	Result	Reference Range
Prothrombin time	16.1 seconds (H)	10.0-12.8 seconds
International normalized ratio	1.4	0.8-1.1
White blood cell	12.9 cells/µL (H)	4.0-10.5 cells/µL
Red blood cell	4.6 x 10^6^/µL	3.93-5.22 x 10^6^/µL
Hemoglobin	12.6 g/dL	11.2-15.7 g/dL
Hematocrit	37.6%	34.1-44.9%
Mean corpuscular volume	81.7 fL	79.4-94.8 fL
Mean corpuscular hemoglobin	27.4 pg	25.6-32.2 pg
Mean corpuscular hemoglobin concentration	33.5 g/dL	32.2-35.5 g/dL
Red cell distribution width	13.9%	11.7-14.4%
Platelet count	368 x 10^3^/µL	150-400 x 10^3^/µL
Mean platelet volume	9.1 fL (L)	9.4-12.3 fL
Absolute neutrophils	10.17 x 10^3^ /µL (H)	1.56-6.13 x 10^3^ /µL
Segmented neutrophils	79.2% (H)	34.0-71.1 %
Lymphocytes	1.10 x 10^3^/µL (L)	1.18-3.74 x 10^3^/µL
Monocytes	1.24 x 10^3^/µL (H)	0.24-0.63 x 10^3^ /µL
Eosinophils	0.23 x 10^3^/µL	0.04-0.36 x 10^3^/ µL
Basophils	0.03 x 10^3^/µL	0.01-0.08 x 10^3^/ µL

During the initial assessment, the patient showed signs of acute metabolic encephalopathy due to severe hypokalemia and elevated liver function tests. Clinical concern for sepsis of unknown origin arose due to marked leukocytosis (leukocyte count of 15,000/µL), prompting admission for further evaluation and treatment.

**Figure 3:**
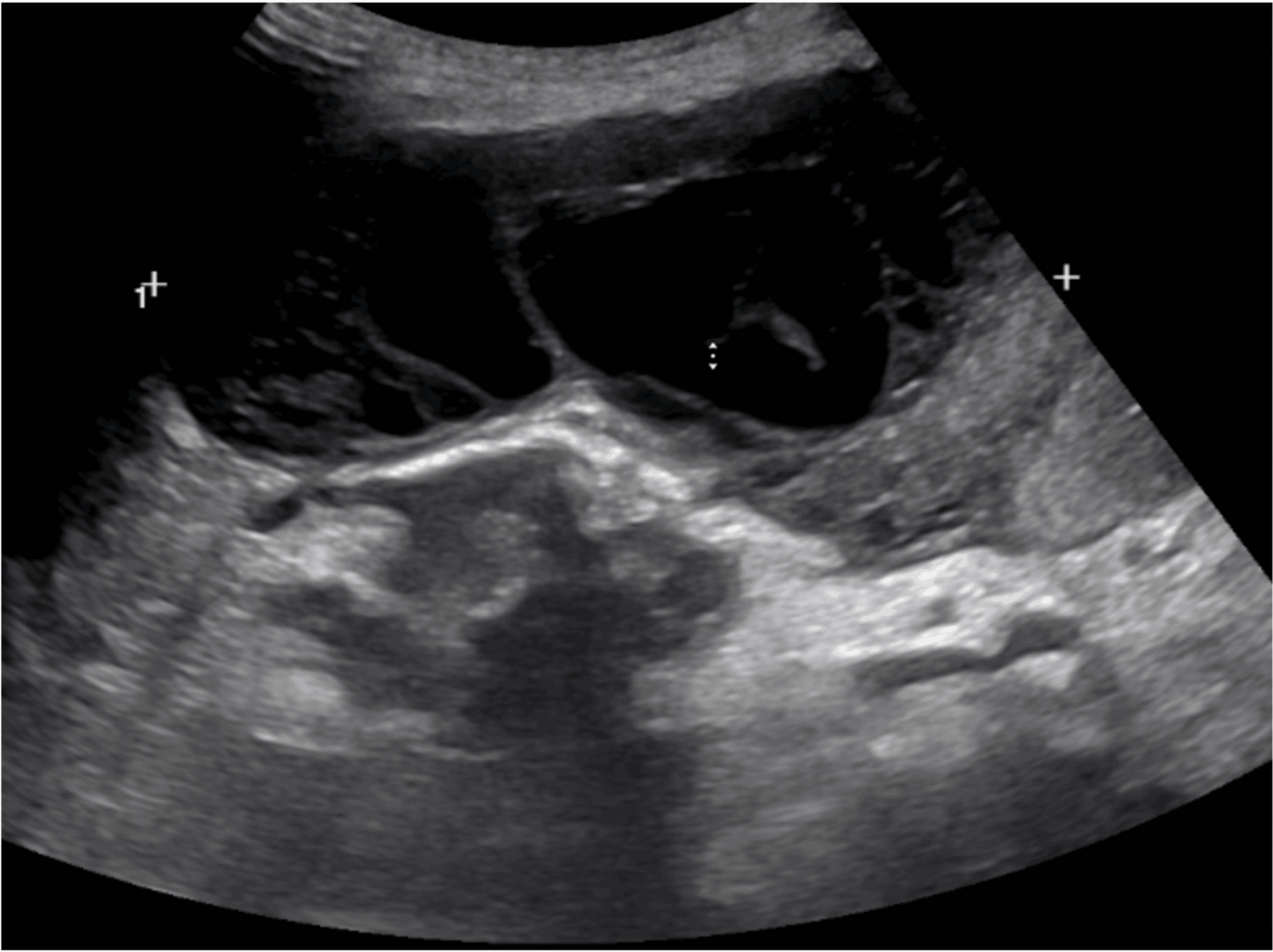
US abdomen. A complex, multiloculated fluid collection is seen anterior to the liver.

A CT angiogram of the chest revealed a 5 mm penetrating aortic ulcer in the proximal descending aorta, which was evaluated by vascular surgery and deemed nonsurgical. Throughout hospitalization, despite the patient not appearing overtly ill (e.g., no signs of respiratory distress or shock), the patient’s labs trended toward worsening sepsis. Leukocytosis progressively increased (from 15,000/µL on admission to 20,000/µL by hospital day 5), as shown in Table 3. C-reactive protein (CRP) and procalcitonin levels remained persistently elevated, and liver function tests revealed mild but worsening transaminitis. Despite broad-spectrum antibiotic coverage, these markers did not improve, prompting additional investigations. Complaints of worsening abdominal pain on hospital day 5 resulted in an abdominal ultrasound, which identified a complex, multiloculated fluid collection anterior to the liver (Figure 3). Gastroenterology was also consulted due to increased failure to thrive and poor nutrition.

**Table 3:** Patient’s White Blood Count (WBC) Trends Since Admission (H)=High value

Day of Admission	WBC (10^3^/uL) (Ref Range 4.0-10.5)
1	12.9 (H)
2	14.0 (H)
3	17.0 (H)
4	16.9 (H)
5	16.3 (H)
6	16.0 (H)
7	12.3 (H)
8	9.6
9	9.9
10	8.9
11	6.4

CT imaging of the abdomen was ordered on day 8 to follow-up on these findings (the delay in completion was due to initial patient refusal), which then confirmed a large bowel obstruction at the level of the sigmoid colon, secondary to significant luminal narrowing. 

**Figure 4:**
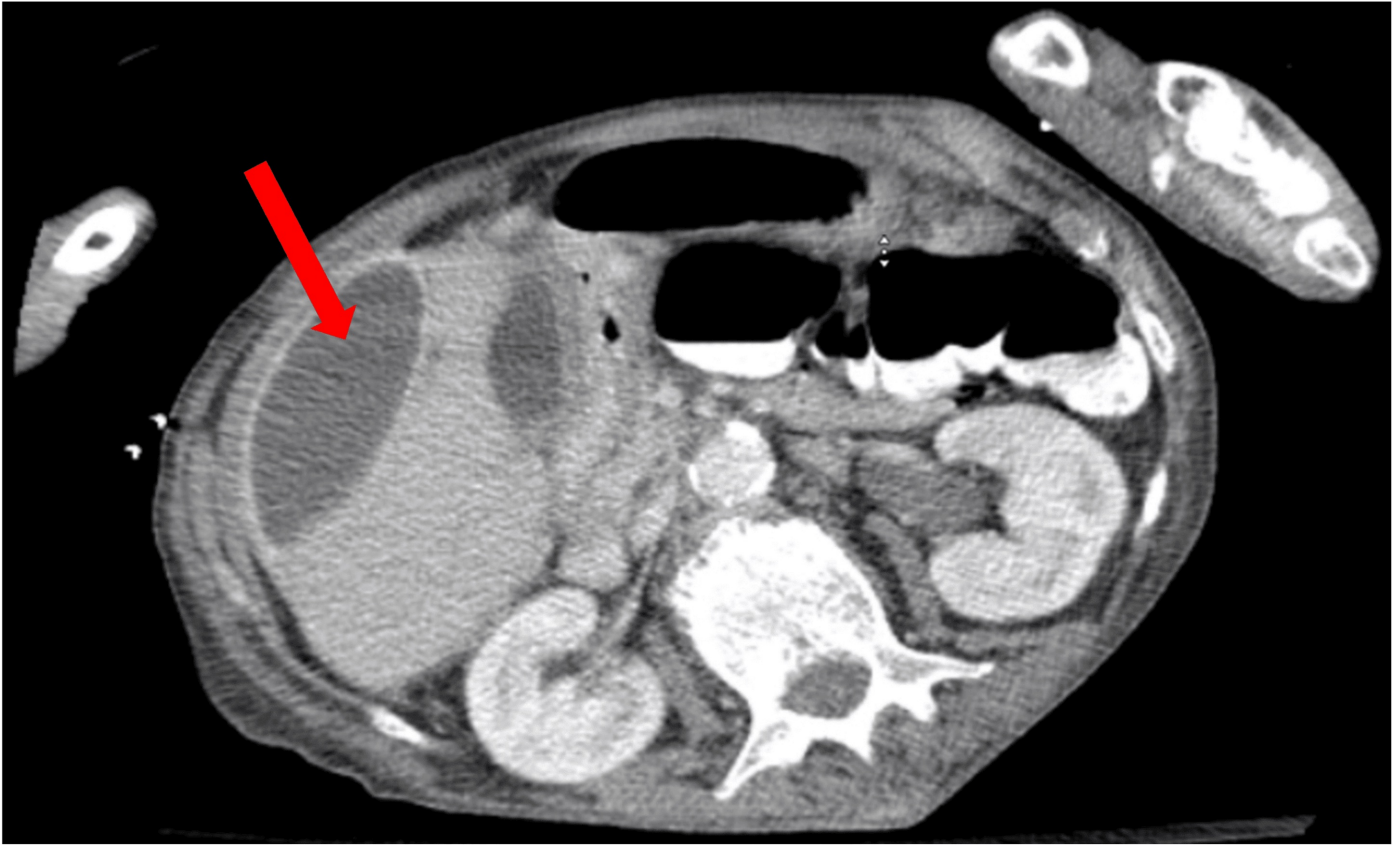
CT abdomen and pelvis prior to drain placement initially interpreted the lesions as large sigmoid bowel obstructions. However, repeat imaging performed two days later accurately identified them as large, multiloculated, interconnected fluid collections, with the largest measuring 16 cm.

**Figure 5:**
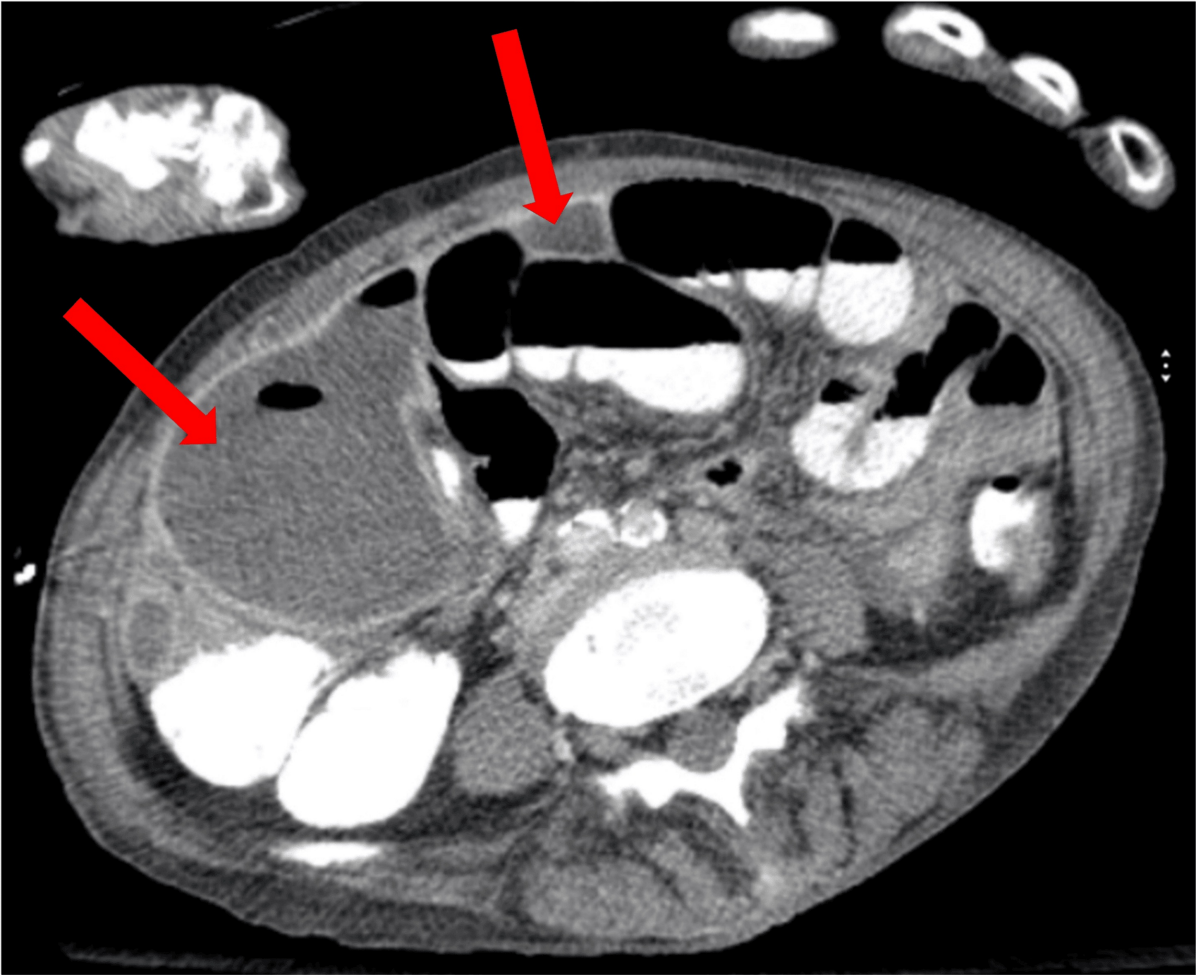
CT abdomen and pelvis prior to drain placement initially interpreted the lesions as large sigmoid bowel obstructions. However, repeat imaging performed two days later accurately identified them as large, multiloculated, interconnected fluid collections, with the largest measuring 16 cm.

**Figure 6:**
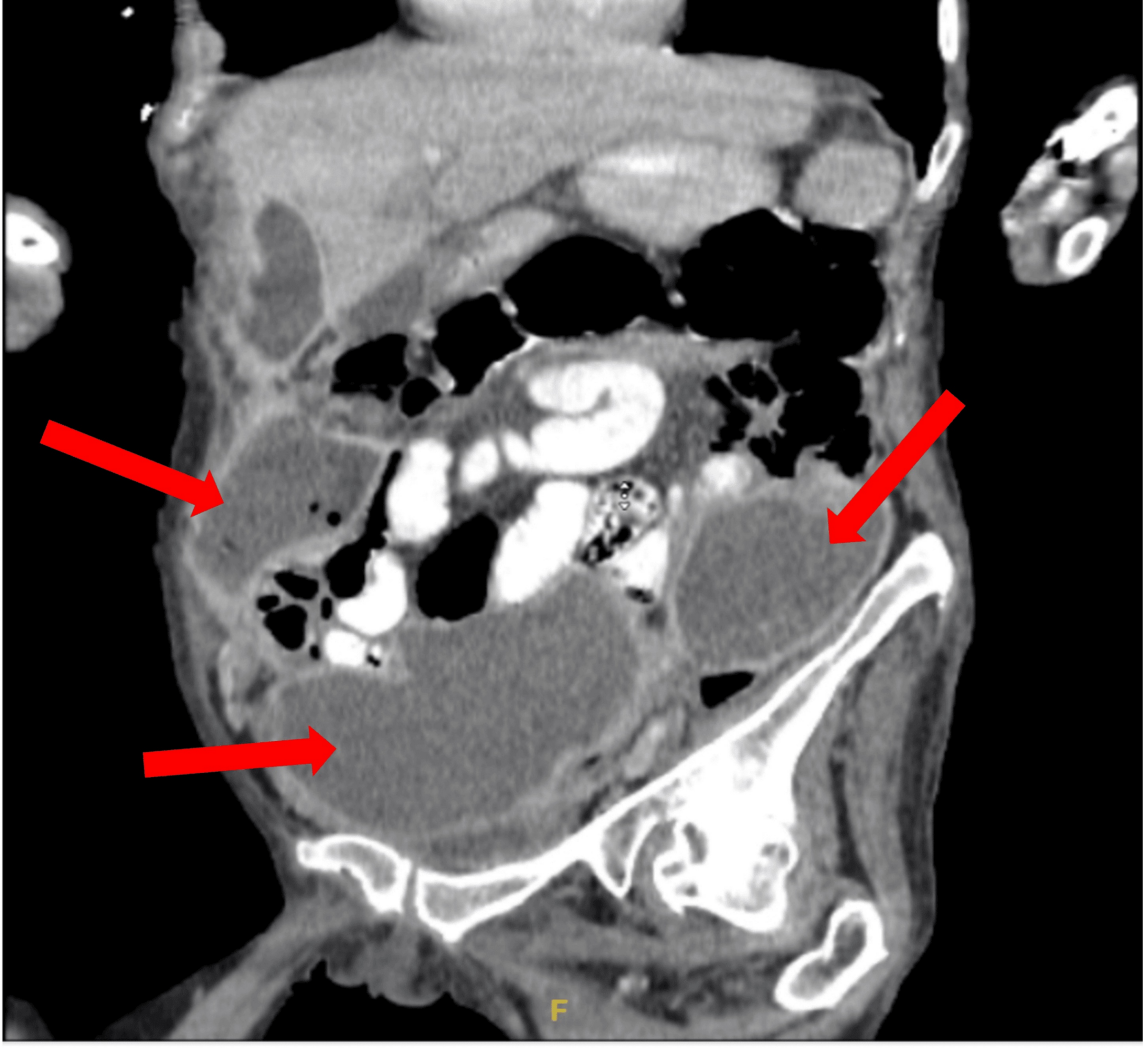
CT abdomen and pelvis prior to drain placement initially interpreted the lesions as large sigmoid bowel obstructions. However, repeat imaging performed two days later accurately identified them as large, multiloculated, interconnected fluid collections, with the largest measuring 16 cm.

The delayed identification of the abdominal abscess likely contributed to the prolonged inflammatory response and the apparent delay in resolving sepsis. The hospitalist and general surgery teams initiated management with intravenous fluids, piperacillin/tazobactam 3.375 g IV every 8 hours and bowel rest. Per the recommendation of Gastroenterology, a flexible sigmoidoscopy was done on hospital day 10 instead of day 8 or 9, as the patient wanted to eat, which delayed the procedure. The study revealed no intraluminal obstructions. Repeat CT imaging on the same day confirmed the presence of multiple large multiloculated abscesses, with the most significant collection on the right side of the abdomen measuring 15 cm in length and midline lower abdomen measuring 16 cm in the transverse direction, as well as a smaller 2.9 cm collection in the left pelvic sidewall (Figures 4, 5, 6).

The patient’s presentation was initially concerning for sepsis of unknown origin, which required consideration of multiple differential diagnoses. Key considerations included IAAs, micro-bowel perforation, and potential urological involvement due to the patient’s presentation with elevated white blood cell count and abnormal imaging findings. Ultimately, abdominal imaging confirmed the presence of multiple abscesses, and cultures grew *Streptococcus anginosus* and* Bacteroides fragilis*, establishing a significant intra-abdominal infection.

The patient underwent ultrasound-guided drainage of the largest abscess on hospital day 11. Interventional Radiology was consulted promptly, and a 12 Fr drain was placed on the same day, aspirating 500 mL of purulent material. Cultures grew *S. anginosus *and *B. fragilis* as seen in Table 4. The patient was continued on piperacillin/tazobactam 3.375 g IV every 8 hours and was administered vancomycin 750 mg IV every 12 hours, along with 125 mg oral vancomycin every 6 hours due to a prior episode of *Clostridioides difficile *during a previous hospitalization. Persistent purulent output was noted on daily follow-up, ranging from 50 to 150 mL per day, with good patient tolerance to drainage procedures.

**Table 4:** Culture Results MIC: Minimal inhibitory concentration; (S)=sensitive.

Organism 1:	*Streptococcus anginosus* (Few)	
Organism 2:	*Bacteroides fragilis* (Abundant)	
*S. anginosus* sensitivities		
Drug	MIC (mcg/mL)	Interpretation
Penicillin	0.047	S
Ceftriaxone	0.50	S
Vancomycin	0.75	S

**Figure 7:**
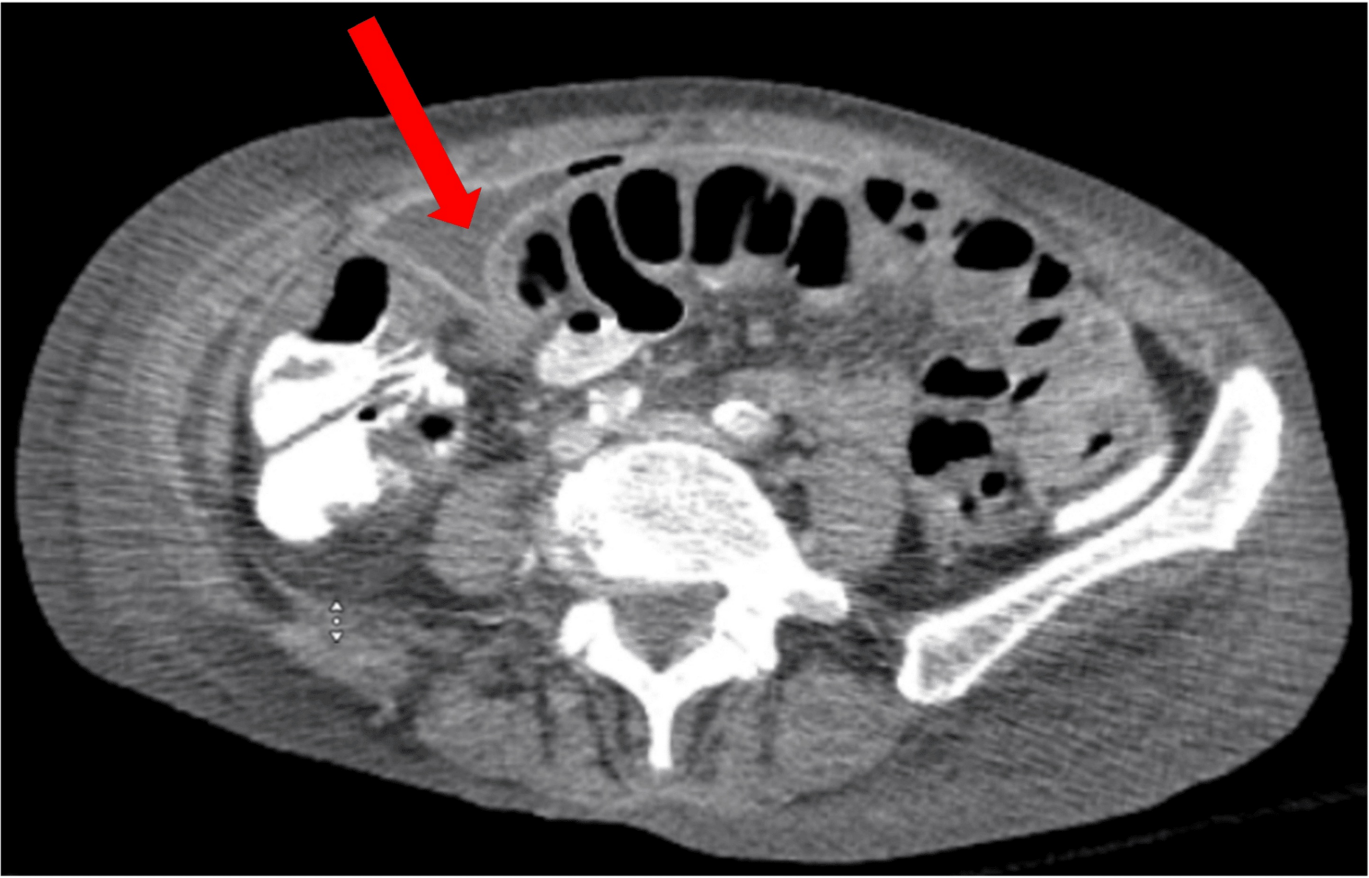
Post-percutaneous drain placement with marked decreased abscess fluid collection.

**Figure 8:**
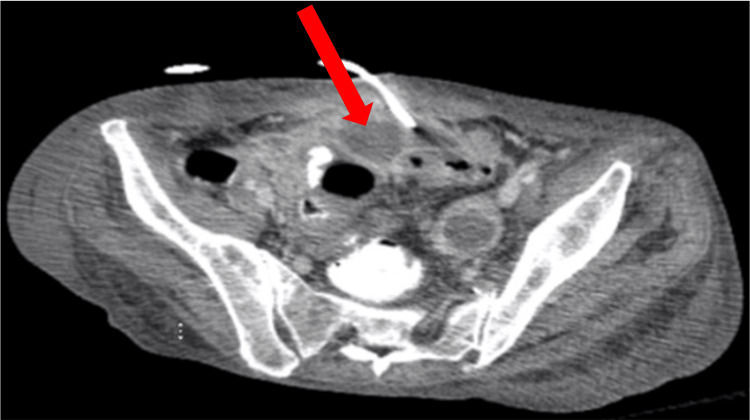
Post-percutaneous drain placement with marked decreased abscess fluid collection.

Repeat CT imaging on hospital day 15 demonstrated a significant reduction in abscess size but suggested a possible bladder communication, evidenced by trace air findings, which could be either due to acute uncomplicated cystitis or recent urethral instrumentation (Figures 7, 8). A CT cystogram was recommended for further evaluation. Despite these findings, the patient remained hemodynamically stable, tolerated a soft diet without abdominal pain, and showed resolution of leukocytosis. Her electrolyte abnormalities were monitored and corrected as needed.

Given the failure to isolate Methicillin-resistant *Staphylococcus aureus *(MRSA), IV vancomycin was discontinued. On hospital day 17, a follow-up CT scan showed further improvement in abscess size, with no definitive evidence of bowel or bladder communication, confirming the absence of a fistulous tract. The patient continued to recover clinically, remaining afebrile with stable vital signs and adequate oral intake. Abscess care is ongoing, with plans for outpatient follow-up and drain removal.

**Figure 9:**
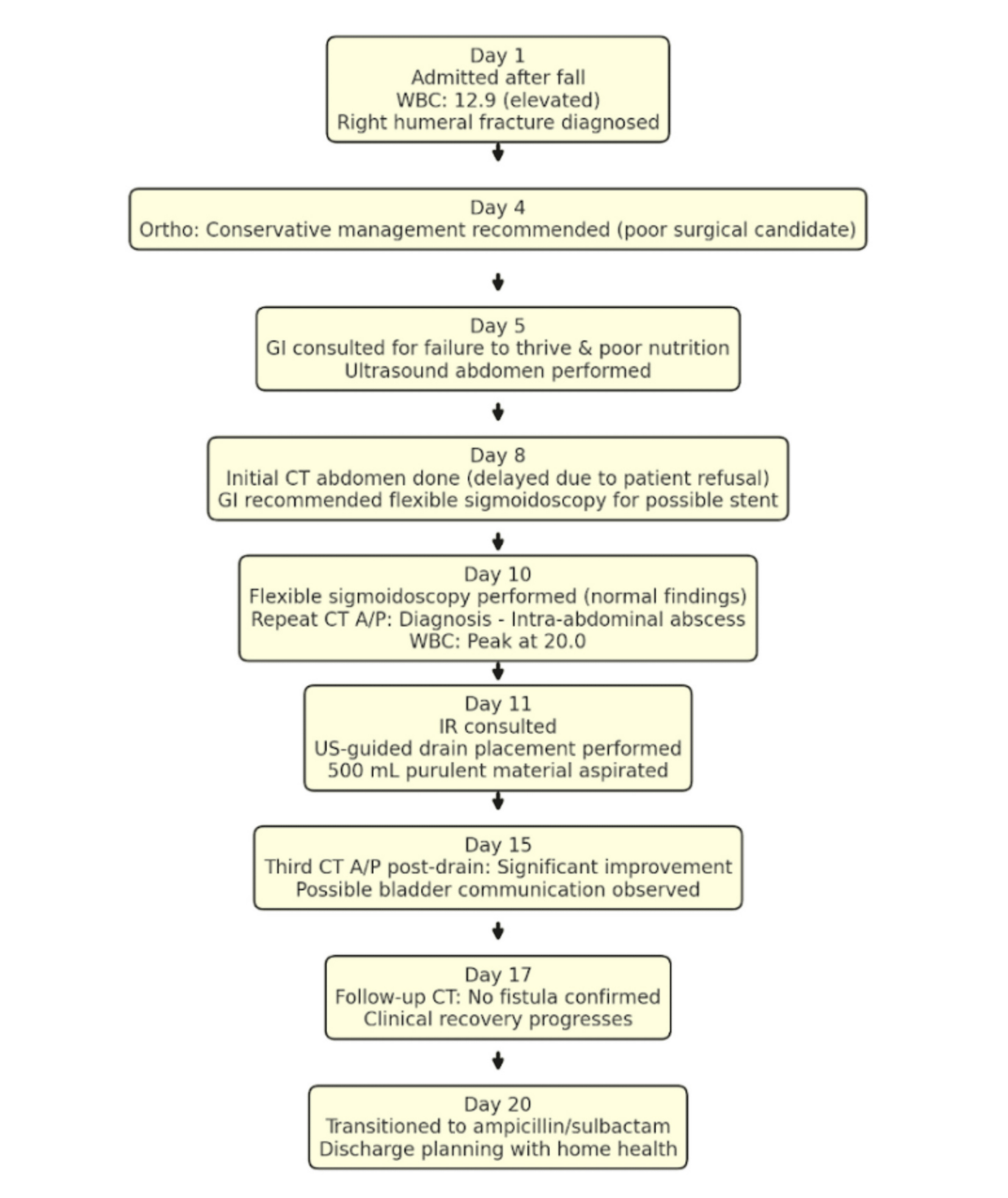
Clinical Timeline from Admission to Discharge WBC: White blood count; CT: computed tomography; A/P: anteroposterior; US: ultrasound; GI: Gasteroenterology consultation

By hospital day 20, the patient showed significant clinical improvement. The hospital team transitioned the patient from piperacillin/tazobactam 3.375 g IV every 8 hours to ampicillin/sulbactam 3 g IV every 6 hours due to continued negative cultures. Discharge plans were coordinated, including home health services for catheter care and outpatient evaluation for potential removal of the drain. The full clinical timeline is summarized in Figure 9.

## Discussion

IAAs, caused by inflammation or disruption of the gastrointestinal tract, can lead to abscesses and are typically polymicrobial. These infections can arise when the mucosal barrier of the gut is breached, allowing normal gut flora to colonizes the abdominal cavity. The most dominant bacteria in intra-abdominal infections are *B. fragilis* and *Escherichia coli*. *B. fragilis* only accounts for 0.5% or less of colonic gut flora but is the most common bacteria isolated in intra-abdominal infections [10,11].

*S. anginosus *is commonly found in abdominal infections and is associated with liver abscesses, intra-abdominal abscesses, and cholangitis. *B. fragilis* and *S. anginosus* were both isolated from the patient's IAA cultures. IAAs infected with *S. anginosus *can be caused by bowel perforation, malignancy, appendicitis, and more [12,13]. However, none of these were explicitly seen in this patient. 

This patient was initially believed to have a colonic distention, suspicious of a large bowel obstruction due to a CT misread, which delayed the real diagnosis. However, throughout her hospitalization, she continued to meet sepsis criteria with worsening leukocytosis, fevers, and tachycardia. On subsequent CT imaging, she was diagnosed with multiple IAAs, the largest on the right side of the abdomen. The cause of these abscesses was also unknown. A colonoscopy ruled out bowel obstruction or any other deformities. However, a micro-perforation is possible due to the patient’s recent history of* C. difficile *[14]. 

Determining the management of intrabdominal abscesses depends on the patient's clinical status. If a patient has a small abscess with no fistula or bowel obstructions, medical management with empiric antibiotics can be used. Once the pathogen is isolated, a more definitive therapy can be used. Percutaneous drains can also be used to drain single unilocular abscesses. For complex abscesses, surgical drains can be placed. The use of medical management and percutaneous drains is a widely used method to avoid surgery. Most patients with complex abscesses require surgery and have a higher mortality risk [15-18].

This patient's care was delayed due to sepsis of unknown origin. It is essential to consider intrabdominal abscesses high on the differential when caring for patients with an unclear clinical presentation.

## Conclusions

Overall, in patients presenting with sepsis of unknown origin and nonspecific symptoms, early imaging is essential to identify abscesses, especially in those with predisposing factors such as recent infections or trauma. IAAs are often polymicrobial, with common pathogens like *S. anginosus* and *B. fragilis *requiring targeted antibiotic therapy. Timely intervention, including imaging-guided drainage and appropriate antibiotics, can effectively manage complex abscesses, reducing the need for surgery. Non-localizing symptoms, as in this case, may remind clinicians to be vigilant from initial clinical interaction and enact an appropriate evaluation with a broad differential diagnosis in septic patients.
